# Development and evaluation of a mobile-optimized daily self-rating depression screening app: A preliminary study

**DOI:** 10.1371/journal.pone.0199118

**Published:** 2018-06-26

**Authors:** Kyungmi Chung, Min-Jeong Jeon, Jaesub Park, San Lee, Chang Oh Kim, Jin Young Park

**Affiliations:** 1 Department of Psychiatry, Gangnam Severance Hospital, Yonsei University Health System, Seoul, South Korea; 2 Department of Psychiatry and Institute of Behavioral Science in Medicine, Yonsei University College of Medicine, Seoul, South Korea; 3 Department of Interaction Science, Sungkyunkwan University, Seoul, South Korea; 4 Division of Geriatrics, Department of Internal Medicine, Severance Hospital, Yonsei University College of Medicine, Seoul, South Korea; Department of Psychiatry and Neuropsychology, Maastricht University Medical Center, NETHERLANDS

## Abstract

The aims of this study were to design a mobile app that would record daily self-reported Korean version of the Center for Epidemiologic Studies Depression Scale-Revised (K-CESD-R) ratings in a “Yes” or “No” format, develop two different algorithms for converting mobile K-CESD-R scores in a binary format into scores in a 5-point response format, and determine which algorithm would be more appropriately applied to the newly developed app. Algorithm (A) was designed to improve the scoring system of the 2-week delayed retrospective recall-based original K-CESD-R scale, and algorithm (B) was designed to further refine the scoring of the 24-hour delayed prospective recall-based mobile K-CESD-R scale applied with algorithm (A). To calculate total mobile K-CESD-R scores, each algorithm applied certain cut-off criteria for a 5-point scale with different inter-point intervals, defined by the ratio of the total number of times that users responded “Yes” to each item to the number of days that users reported daily depressive symptom ratings during the 2-week study period. Twenty participants were asked to complete a K-CESD-R Mobile assessment daily for 2 weeks and an original K-CESD-R assessment delivered to their e-mail accounts at the end of the 2-week study period. There was a significant difference between original and mobile algorithm (B) scores but not between original and mobile algorithm (A) scores. Of the 20 participants, 4 scored at or above the cut-off criterion (≥13) on either the original K-CESD-R (*n* = 4) or the mobile K-CESD-R converted with algorithm (A) (*n* = 3) or algorithm (B) (*n* = 1). However, all participants were assessed as being below threshold for a diagnosis of a mental disorder during a clinician-administered diagnostic interview. Therefore, the K-CESD-R Mobile app using algorithm (B) could be a more potential candidate for a depression screening tool than the K-CESD-R Mobile app using algorithm (A).

## Introduction

Recently, rapid growth in the use of mobile devices, such as smartphones, tablet PCs, and smartwatches, has prompted people to readily report daily mental health ratings via mobile apps as an ecological momentary assessment tools, highlighting the clinical importance of the temporal dynamics of depression and other mood states [1–6]. By reviewing daily self-reported data on currently experienced symptoms together in an outpatient setting, both patients and healthcare practitioners can devote valuable time to talking about event-related mood fluctuations within specific time windows. Despite some concerns on using simpler and shorter depression screening tools [7] designed for smartphone users, a shorter recall period not only reduces measurement errors and helps users remember events triggering depressed mood but also offsets limitations of traditionally administered (i.e., paper-and-pencil-based or web-based) or revised short-form scales [3]. Moreover, potential benefits of the development of a mobile-optimized depression screening app can be maximized based on key features of smartphone apps such as their storage, portable accessibility, and time-sensitive local and push notifications [1].

In general, most well-established depression screening tools, such as the Beck Depression Inventory (BDI [8]), Patient Health Questionnaire-9 (PHQ-9 [9]), Center for Epidemiologic Studies Depression Scale-Revised (CESD-R [10]), Zung Self-Rating Depression Scale [11], and Geriatric Depression Scale (GDS [12]), have some limitations. First, these scales, which meet the 5th edition of the American Psychiatric Association’s Diagnostic and Statistical Manual of Mental Disorders (DSM-5) criteria for depression [13], are administered at fixed time points, asking participants or patients to report the severity of depressive symptoms experienced over the past 2 weeks. As memory decay increases with longer recall periods and less salient events [14], diagnosis based on retrospective recall is vulnerable to potential measurement errors. Secondly, 4- or 5-point scales are not positioned at equidistant intervals, particularly the distance between 2 and 3 points versus that between 3 and 4 points (e.g., 0 = “Not at all or less than one day,” 1 = “1–2 days,” 2 = “3–4 days,” 3 = “5–7 days,” and 4 = “Nearly every day for 2 weeks”). This is because the 4-point response category was added after the 3-point response category at a later time point to reflect current diagnostic criteria for major depression [15]. Finally, 4- or 5-response option formats written in longer sentences (e.g., 0 = “I get as much satisfaction out of things as I used to,” 1 = “I do not enjoy things the way I used to,” 2 = “I do not get real satisfaction out of anything anymore,” and 3 = “I am dissatisfied or bored with everything”) may increase administration time and response burden in the young or elderly [7, 16, 17]. These limitations could be overcome by developing a new daily self-rating depression scale for both general and patient populations.

Despite the need for a new concept of depression screening scales, few scientific studies have investigated the use of daily self-reporting mobile apps using the PHQ-9 with a binary- or 4-response option [2, 3], Although the PHQ-9 has been used to develop mobile apps for depression screening [2, 3, 18], it has been more difficult to search for and find CESD-R apps than PHQ-9 apps at Google Play and the Apple App Store. Compared with the BDI, the CESD-R has been more widely used in the field of psychiatric epidemiology in both general and patient populations and is available free of charge for research purposes. Moreover, the CESD-R has been tested for its reliability, validity, and psychometric properties in Korean healthy individuals and patients with depression [19]. For these reasons, the Korean version of the CESD-R (K-CESD-R) scale was chosen for this study.

The purposes of this study were to: (1) design a mobile app that gathers daily self-reported K-CESD-R ratings in a simple binary (i.e., “Yes” or “No”) format, (2) develop two different algorithms for converting total mobile K-CESD-R scores in binary format ranging from 0 to 20 into scores in a 5-category format ranging from 0 to 80, (3) compare an original version of the K-CESD-R (i.e., created and conducted via an online survey tool; hereafter “online”) and two mobile K-CESD-R versions in a sample of younger adults, and (4) determine which algorithm (A vs. B) is more appropriately applied to the newly developed app as compared with diagnostic consultations with a clinician.

## Methods

### Participants

Participants were recruited via advertisements posted on university websites in Seoul, South Korea. Online advertisements contained a link to the sub-investigator’s e-mail to enable volunteers to send their application forms. From the volunteer pool, which consisted of healthy individuals who met inclusion and exclusion criteria, 20 undergraduate students (9 women; aged 19–29 years; *M* = 22.20, *SD* = 2.69) were enrolled after providing signed informed consent. Individuals under 19 years of age, with any history of neurological or psychiatric disorders, or without their own smartphone with a screen size of at least 4 inches diagonally were not eligible to participate in this study. Of the 20 participants, 13 were iPhone users (65%), and seven were Android phone users (35%). The study was approved by the Institutional Review Board (IRB) of Gangnam Severance Hospital. All participants were paid KW 30,000 for their participation.

### Development of the K-CESD-R mobile app

The K-CESD-R [19], which has 20 items rated from 0 to 4 with 80 as the total possible score, was chosen to develop a new version of the K-CESD-R scale (using a cut-off point ≥13) optimized for the current mobile environment.

### User interface (UI) and graphical user interface (GUI) design

**Fig 1:**
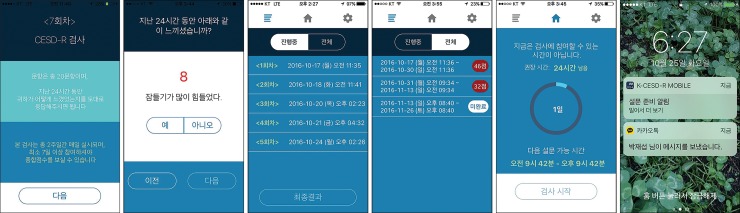
Screenshots of UI/GUI design of the K-CESD-R Mobile app.

Apple iOS and Android smartphone apps were developed with the same UI and GUI. The app was designed to (1) guide users to respond to a “Yes” or “No” response option version of the K-CESD-R based on their experiences over the past 24 hours, (2) administer a session every day for 2 weeks, and (3) measure response times (RTs) to pressing a “Yes” or “No” button after each item was presented (Fig 1).

The 20 items were presented in a randomized order to control for an order effect. To improve adherence rate, users could freely start the next session any time within a time window of ± 6 hours from the recommended time of 24 hours after completing the previous session as displayed on the K-CESD-R Mobile app home screen. Users were prompted to complete the day’s session with a maximum of three local notifications (i.e., 24, 26, and 28 hours after completing the previous session). Users could choose to be reminded of the remaining time available to complete the next session through scheduled local notifications visible in the Apple iOS notification center/Android notification panel so that they could start the session at an appropriate time without disruption to their everyday life. To ensure accuracy of measurement, users were particularly encouraged to complete the session in the recommended time when the first notification was delivered to their mobile phones. After the 6-hour time window from the recommended time, the session was closed.

On the home screen page of the app, users could check three pieces of information until the waiting period expired: the day count, the estimated time remaining to the recommended completion time of the next session, and the entire time window when the next session would be available. To display this information on the page, all entry and administration times for the 20 items were recorded. In the main “History” menu represented by a 4-line hamburger icon, users could review the day’s responses in “Yes” or “No” format on a “In progress” tab during the 2-week study period. On the last day of the study period, the final score was computed when users pressed a “Final result” button, which appeared only if users completed at least seven daily sessions. Otherwise, a “Finish” button appeared. On an “Entire” tab, users could review a list of all complete and incomplete session results, either scored from 0 to 80 or marked as “Incomplete,” and a brief interpretation of the results.

#### Algorithms

**Fig 2:**
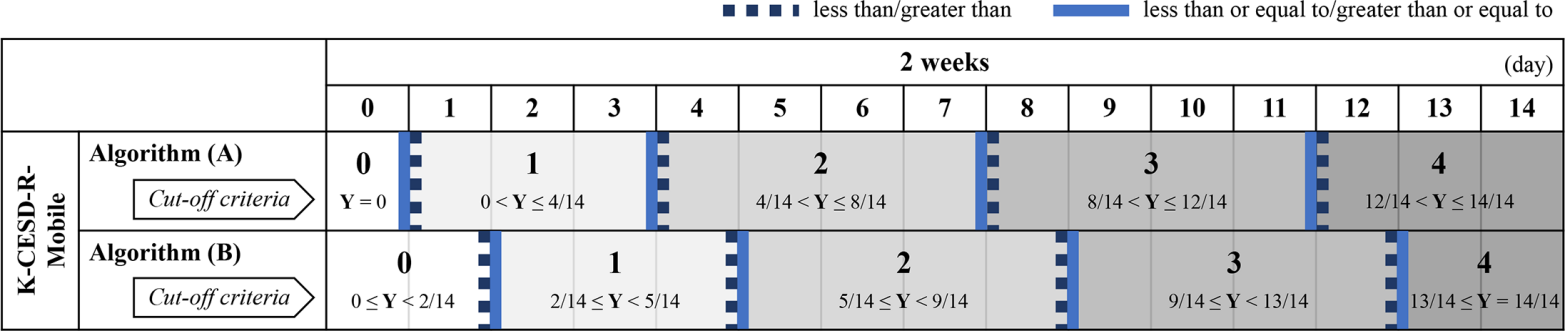
Illustration of response conversion from the binary response format of the mobile K-CESD-R into a 5-point response format. Unlike the standard online K-CESD-R based on a “frequency” approach, the K-CESD-R Mobile app employed two different algorithms based on a “ratio” approach to deal with missing data in prospective daily assessment of depressive symptoms across the 2-week study period. Y = Q/P (Q = total number of times users responded “Yes” to each item; P = total number of days that users completed sessions during the 2-week study period).

If users completed fewer than seven daily sessions during the 2-week study period, their data were excluded from further analysis. To estimate the severity of users’ depressive symptoms during the 2-week study period, simple binary response data from a newly developed mobile K-CESD-R scale were compared with 5-point response data from the online K-CESD-R scale. All “Yes” responses to each of the 20 items were summed and recoded using two different converting algorithms (Fig 2).

Algorithm (A) focused on improving the scoring system of the online K-CESD-R scale. A response was scored as “0” when a symptom lasted for “less than 1 day last week,” which could be interpreted as “no symptom at all.” A response was scored as “1” if users answered “Yes” at least once during the 2-week study period. Using this algorithm, scores were more likely to be higher than expected due to a low threshold. Algorithm (B) focused on further refining the scoring system of the mobile K-CESD-R scale from algorithm (A). As the cut-off value for the K-CESD-R (i.e., 13) was set lower than that of the original CESD-R (i.e., 16), scores at the boundaries of ranges were included in the higher range in the 5-point scoring system. For example, if users responded “Yes” five times in the last 14 days, this corresponded to 2.5 times a week, which would be located between “1” (1–2 days) and “2” (3–4 days) in the original CESD-R. Therefore, algorithm (B) for the mobile K-CESD-R would score this case as “2.” This scoring system was applied to the remaining “3” (5–7 days) and “4” (nearly every day for 2 weeks) in the same manner. With these two algorithms, the severity of depressive symptoms could be immediately estimated on a daily basis, which has advantages over retrospective recall-based evaluation.

#### Database security, storage and authorization

After users signed up for the K-CESD-R Mobile app, their unique personal information, such as gender, date of birth, and mobile phone number, was de-identified to ensure confidentiality by assigning an identification code to each user. For data analysis, only the demographic characteristics of gender and age were used.

When users completed the day’s session and pressed the “Save and Send” button, data were uploaded from the users’ mobile phones and stored on an Amazon Web Services server. It was possible to remotely monitor each user’s progress and completion rate and download a locked data file on a web dashboard, to which access was limited to authorized researchers provided with a master ID and a password for sign-in and an additional password to unlock the raw file for the personal information protection.

### Procedure

For their initial participation, participants gathered in the grand auditorium at Gangnam Severance Hospital on the same day and time. After providing informed consent to experimenters registered as qualified clinical research coordinators, participants were asked to complete a pre-questionnaire to obtain demographic data. To prevent non-participants’ mental health data from being collected without their consent, enrolled participants who met inclusion and exclusion criteria were registered as beta testers for the study period. Following the experimenters’ instructions, Android phone users could directly search for and download the beta app, “K-CESD-R Mobile,” on the Google Play store, whereas iPhone users could download the same app only via the Apple’s TestFlight platform for beta testing. To address security and privacy issues surrounding personal and sensitive information collected and transmitted via mobile devices, the K-CESD-R Mobile app obtained participants’ consent to collect and use their information for the purpose of research under Korea’s personal information protection act. After accepting all terms of use and acknowledging the privacy statement, participants were allowed to create an account and start a new session. Following session completion, participants were informed that additional sessions should be completed every day for 2 weeks and that an online K-CESD-R assessment using Survey Monkey (www.surveymonkey.com) would be delivered via e-mail at the end of the 2-week study period. Therefore, both mobile and online assessments should be completed by the final day of the study period.

After analyzing differences between algorithms (original vs. mobile algorithm A vs. mobile algorithm B), participants who scored ≥13 were guided to visit a psychiatry outpatient clinic at the hospital for further diagnostic consultations. For approximately 30–40 min, a medical doctor in the Department of Psychiatry conducted structured interviews, such as the original English version of Clinical Global Impressions-Severity of Illness Scale (CGI-S [20]) and Korean versions of the Montgomery-Asberg Depression Rating Scale (K-MADRS [21, 22]), Hamilton Anxiety Rating Scale (K-HAM-A [23, 24]), Hamilton Depression Rating Scale (K-HAM-D [25, 26]), and Mini-International Neuropsychiatric Interview (M.I.N.I. Korean version 5.0.0. [27, 28]). No additional follow-up visits were required.

### Measurement instruments

#### Self-report depression screening scales

PHQ-9. Based on nine DSM-IV depression diagnostic criteria, the PHQ-9 [9] has 9-item depressive symptom modules scored from 0 (not at all) to 3 (nearly every day) and asked participants to self-report any of the experienced symptoms that bothered them over the last 2 weeks. Total PHQ-9 score could range from 0 to 27. According to the scoring and interpretation guide of the PHQ-9, participants with a score ≥10 were considered as being in need of further follow-up and treatment, pharmacotherapy, and psychotherapy. This study administered the validated Korean version of the PHQ-9 [29].

K-CESD-R. The CESD-R [10] is composed of 20 items defined by DSM-IV criteria. The 20 items were rated on a 5-point scale from 0 (not at all or less than 1 day) to 4 (nearly every day for 2 weeks). Total scores for the original CESD-R and the K-CESD-R [19] could range from 0 to 80. The cut-off score of the K-CESD-R was three points lower than that of the CESD-R (i.e., ≥16).

#### Clinical diagnostic interview scales

CGI-S. The severity of illness was estimated with the CGI-S [20], which is rated on a 7-point scale using a range of responses from 1 (normal, not at all ill) to 7 (among the most extremely ill).

K-MADRS. The K-MADRS [22] is the Korean version of the MADRS [21], a 10-item clinical rating scale for measuring the severity of depressive symptoms. Each item was rated on a scale from 0 to 6, and total score could range from 0 to 60. The diagnostic threshold for depression was a score of ≥16.

K-HAM-A and K-HAM-D. The HAM-A [23] and HAM-D [25] were used to evaluate the severity of anxiety and depression, respectively. In this study, the Korean versions of the HAM-A [24] and HAM-D [26], both of which have acceptable reliability and validity, were utilized. For the K-HAM-A, each item was scored on a scale from 0 (not present) to 4 (severe), with a total score >25 indicating moderate to severe anxiety. For the K-HAM-D, the total score was the sum of scores from the first 17 items, with a cut-off score of ≥19.

M.I.N.I. The M.I.N.I. 5.0.0. [27] is a short, structured diagnostic interview containing different modules identified by letters, each corresponding to a diagnostic category. According to DSM-IV and the 10^th^ edition of the International Classification of Diseases (ICD-10) criteria, psychiatric disorders were diagnosed with time frames. In this study, the Korean version of the M.I.N.I. 5.0.0. [28] was used.

## Results

### Comparison of online and two mobile K-CESD-R scores computed with different algorithms

To compare an online K-CESD-R (i.e., prediction: a 2-week delayed recall test) and two mobile K-CESD-R (i.e., observation: a 24-hour delayed recall test for 2 weeks) scores, paired *t*-tests were separately performed, and the mean absolute error (MAE) between predicted and observed values was calculated as follows: (1) online vs. mobile algorithm (A) and (2) online vs. mobile algorithm (B). No significant difference between online and mobile algorithm (A) scores was found (*t* (19) = -1.192, *MAE* = 1.550, *p* = .248, two-tailed). However, there was a significant difference between online and mobile algorithm (B) scores (*t* (19) = 3.023, *MAE* = 2.500, *p* < .01, two-tailed). Compared with the online K-CESD-R score (*M* = 5.85, *SE* = 1.24), the mobile algorithm (A) score (*M* = 6.40, *SE* = 1.02) tended to overestimate the severity of depressive symptoms, whereas the mobile algorithm (B) score (*M* = 4.15, *SE* = .85) underestimated the severity of symptoms. High correlations were observed between the online K-CESD-R total score and scores from the mobile K-CESD-R converted with algorithm (A) and (B) (*r* = .934 and .920, *p* < .001, respectively).

### Concurrent validity of K-CESD-R scores with PHQ-9 score

As another depression screening tool for which no permission is required to reproduce, translate, display, or distribute, the PHQ-9 has been most frequently applied to the development of smartphone-based depression screening apps. Concurrent validity was assessed by correlating PHQ-9 total score with the total scores of the online K-CESD-R (*r* = .444, *p* = .050) and the two mobile K-CESD-R computed with algorithm (A) (*r* = .430, *p* = .059) and algorithm (B) (*r* = .343, *p* = .139). Moderate positive correlations were found between the PHQ-9 score and all three K-CESD-R scores.

### Agreement between K-CESD-R scores and clinical assessment

To clarify which of the two versions of mobile K-CESD-R would show potential as an adequate substitute for the online K-CESD-R to screen for depression for clinical purposes, clinical assessments with the CGI-S, K-MADRS, K-HAM-A, K-HAM-D, and M.I.N.I. Korean version 5.0.0. were conducted if any one of the three K-CESD-R scores was ≥13. Out of 20 participants, 4 and 3 had scores ≥13 on the online K-CESD-R and mobile K-CESD-R converted using algorithm (A), respectively. When converted using algorithm (B), only one participant had a score of ≥13 (Table 1). The total scores of all clinical interview scales for these four participants (i.e., P4, P5, P17, and P20) were below the diagnostic thresholds for mental disorders as judged by the CGI-S (≥3), K-MADRS (≥16), K-HAM-A (≥25), K-HAM-D (≥19), and M.I.N.I. (based on modules that met DSM-IV diagnostic criteria for a major depressive episode).

**Table 1:** List of all participants’ K-CESD-R scores. Asterisks (*) indicate scores that met the cut-off criterion (≥ 13).

Participant ID	Online	Mobile algorithm (A)	Mobile algorithm (B)
**P1**	2	2	0
**P2**	2	3	3
**P3**	6	5	3
**P4**	13*	11	9
**P5**	16*	13*	9
**P6**	3	6	2
**P7**	3	6	4
**P8**	3	7	6
**P9**	1	1	0
**P10**	3	5	1
**P11**	0	0	0
**P12**	0	5	2
**P13**	2	3	3
**P14**	7	8	3
**P15**	2	1	0
**P16**	3	3	2
**P17**	17*	16*	13*
**P18**	8	9	5
**P19**	12	10	8
**P20**	14*	14*	10

To estimate the degree of inter-rater reliability and determine which algorithm better predicted the result of clinical assessment, the online K-CESD-R and two mobile K-CESD-R scores were recoded into nominal scale data based on a cut-off score of 13: either 1 (non-depressed) or 2 (depressed). According to the results of structured diagnostic interviews reviewed and interpreted by a licensed clinician in the Department of Psychiatry, participants were classified as either 1 (non-depressed) or 2 (depressed). The highest percent agreement with clinical assessment was observed for the mobile K-CESD-R computed with algorithm (B) (95%), followed by the mobile K-CESD-R computed with algorithm (A) (85%) and the online K-CESD-R (80%) (Table 2). Inter-rater reliability as measured by Scott’s Pi further revealed that all observed agreements were lower than expected agreements, as all values of Scott’s Pi were negative. Compared with clinical assessment, the two mobile K-CESD-R showed smaller differences between expected and observed agreements than the online K-CESD-R. Furthermore, the difference was the smallest for the comparison of the mobile K-CESD-R with algorithm (B) and clinical assessment.

**Table 2:** Inter-rater reliability between the self-rated K-CESD-R scales and a clinician-administered diagnostic interview.

	N of agreements	N of disagreements	Percent agreement (%)	Scott’s Pi (Nominal)		
				Value of Scott’s Pi	Expected agreement	Observed agreement
**Online—CA**	16	4	80	- .111	.820	.800
**Mobile (A)—CA**	17	3	85	- .081	.861	.850
**Mobile (B)—CA**	19	1	95	- .026	.951	.950

Online, online K-CESD-R; Mobile (A), mobile K-CESD-R with algorithm (A); Mobile (B), mobile K-CESD-R with algorithm (B); CA, clinical assessment.

### Administration time and adherence rate

Based on RT data, the average administration times (i.e., overall RTs taken to respond to 20 items) for the 2-week study period were calculated. Administration times ranged from 10.397 to 115.081 s in this sample of younger adults (*M*_*total*_ = 25.157, *SE*_*total*_ = 4.942). The adherence rate for the K-CESD-R Mobile app was 100% across the 2-week study period.

## Discussion

To our knowledge, this is the first report on the development and evaluation of a mobile-based daily self-rating depression screening tool with K-CESD-R items in a different response format. In this preliminary study, we hypothesized that the K-CESD-R Mobile app would keep track of day-to-day variability in depressive symptoms, help screen for depression in the early stage by reducing potential measurement error, and lead people at risk for depression to visit a psychiatry clinic for consultation on their mental health concerns. Researchers and mental health practitioners are likely to share a common interest in increasing the true-positive rate (i.e., sensitivity) and decreasing the false-positive rate (i.e., specificity) for self-reports of depression. In attempts to identify people with or without depression, two algorithms were employed for recoding binary responses (0 = No, 1 = Yes) into 5-point responses and calculating mobile K-CESD-R scores, corresponding to online K-CESD-R scores ranging from 0 to 80. Further clinician-administered assessment was also carried out for participants who screened positive for depression on either online or mobile K-CESD-R scales. These two steps allowed us to draw the careful conclusion that algorithm (B) might be more appropriate than algorithm (A) for use in this newly proposed, mobile-optimized daily self-rating depression screening app. Moreover, the significant difference between scores on the online K-CESD-R and the mobile K-CESD-R computed with algorithm (B) suggests that the online K-CESD-R adopting a 5-point response option format might be a less accurate reflection of currently experienced depressive symptoms based on blurred memories over the last 2 weeks than the mobile K-CESD-R, which is a 24-hour recall test adopting a binary response option format.

In contrast to the retrospective recall-based CESD-R, the K-CESD-R Mobile app utilized a ratio approach to handle missing data if users responded to the assessment items for at least 7 days in the 2-week study period. By compensating for the frequency approach of the original K-CESD-R, two conversion algorithms had different interval durations defined by the ratio (i.e., the total number of times users responded “Yes” out of the total number of days in which users reported daily ratings during the 2-week period), so that the difference between each point resulted in upward adjustment in algorithm (A) and resulted in downward adjustment in algorithm (B). Furthermore, previous studies report that mobile phone-captured data might be more sensitive than paper-and-pencil-collected data, as participants feel more comfortable reporting their experienced depressive symptoms via mobile apps installed on their own smartphones [2, 30, 31]. By considering and addressing this issue, this study additionally focused on the dynamic nature of depression, particularly suicidality. Surprisingly, few participants whose total scores were <13 reported suicidal thoughts via the app, and none whose scores were ≥13 reported suicidal ideation. Whereas the aim of several previous studies was to estimate the optimal cut-off points of depression screening instruments based on longer periods of retrospective recall, the K-CESD-R Mobile app can obtain information on intra-individual depressive symptoms and their variations more sensitively and accurately than the online K-CESD-R scale. However, in line with previous and present findings, depression screening data collected from the K-CESD-R Mobile app should be carefully interpreted depending on the participant sample. Given that the latest Organisation for Economic Co-operation and Development (OECD) data from 2017 [32] show that South Koreans suffer from a lack of sleep and that average annual hours worked are the third-longest among OECD member countries, it could be assumed that regardless of the severity of depressive symptoms, Koreans would be more likely to be sensitive to the three sleep-related and two fatigue-related items, thus raising the possibility of overemphasizing their hectic life patterns.

More importantly, the feasibility and effectiveness of Korean epidemiologic, momentary mental health data collection can be considered as key features of this new depression screening app. Despite the limitation of the small sample size in the present study, participants demonstrated a 100% adherence rate over the 2-week study period, and there were no missing data from daily self-reports of depressive symptoms. Compared with systematic reviews reporting comparatively low levels of adherence to the use of newly developed mental health apps (i.e., ranging from 65% to 91% [2, 33–35]), the K-CESD-R Mobile app had the highest adherence rate. A possible explanation is that, in the process of recruiting participants, the purpose of this study (including the difference between the 2-week delayed retrospective recall-based online K-CESD-R and the 24-hour delayed prospective recall-based mobile K-CESD-R scales) were explained to all participants. As a result, they may have been motivated to complete the two different versions of the assessment to provide more accurate results. Despite the potential impact of the Hawthorne effect on our results, the possibility that the K-CESD-R Mobile app itself or participants’ own needs to monitor their mental health conditions encouraged them to comply with the local notification delivered via the app or the guidance provided by the experimenters cannot be ruled out, as all four participants whose online or mobile K-CESD-R scores were ≥13 took part in the clinician-administered diagnostic interview. However, according to a meta-analysis of the effect of depression on patient adherence, depression is more of a risk factor for poor adherence to treatment recommendations compared with anxiety [36]; therefore, users or patients with mild to severe depressive symptoms would be more likely to be non-compliant with medical devices and mental healthcare apps [37]. Considering the comorbidity of depression and other mental disorders or medical diseases, the K-CESD-R Mobile app not only serves as an effective and valid tool for collecting daily depressive symptoms in general populations, but future studies utilizing this app should also explore the potential for mobile interventions in those with depression.

As another key feature of the K-CESD-R Mobile app, RTs were measured for all 20 items even though both the PHQ-9 and CESD-R are based on Classical Test Theory, which focuses on the reliability of psychological tests and the true score (i.e., the observed score determined by the actual state of individuals’ unobservable symptoms). Even if participants paused or hesitated, they could complete the session within approximately 2 min, based on the rationale of “ease of use” and “reduced cognitive demand.” Considering that the average administration time of elderly persons ranges from 7 min to as long as 10–12 min [16], the K-CESD-R Mobile app has the potential to be substituted for the 30-item GDS with a binary response format, which was developed for use in geriatric populations. In this preliminary study, the relationship between total score and RT was not considered. However, based on a self-schema model for depression [38], an “inverted-U effect” will be investigated with a sufficiently large sample size and a revised version of the K-CESD-R Mobile app in future studies.

Assuming that this app is designed to record personal lifetime history of depressive episodes, there are some limitations to the generalizability of the study findings. The first limitation is that the small sample size may have led to higher variability and thus lower reliability of study results. Furthermore, different populations are unlikely to respond in the same manner to each item of the K-CESD-R. To cope with the second limitation, along with the aforementioned sample size problem, the use of an epidemiologic depression screening tool across a wide range of general and clinical populations with different self-schemata for depression in terms of content and efficiency [38] should be investigated in future studies. Finally, the stability of adherence rates over time should be examined to determine whether the K-CESD-R Mobile app can motivate users to continue its use for a prolonged period of time (e.g., at least 4 weeks for test-retest reliability).

## Conclusion

Despite its limitations, this preliminary investigation suggests that a K-CESD-R Mobile app utilizing algorithm (B) is more predictive than that utilizing algorithm (A) based on an assessment of inter-rater reliability between three self-rated K-CESD-R scales and a clinician-administered diagnostic interview. The possibility that the K-CESD-R computed with algorithm (B) is more reflective of objective scoring criteria will be more thoroughly explored in future research.

## Supporting information

S1 File Data set of this study. (XLSX)
